# Radiomics optimizing the evaluation of endometrial receptivity for women with unexplained recurrent pregnancy loss

**DOI:** 10.3389/fendo.2023.1181058

**Published:** 2023-08-08

**Authors:** Wendi Huang, Yi Jin, Lulu Jiang, Mengjie Liang

**Affiliations:** Department of Ultrasound Imaging, The First People’s Hospital of Wenling, Wenling, Zhejiang, China

**Keywords:** radiomics, recurrent pregnancy loss, endometrial receptivity, superfertile, ultrasound

## Abstract

**Background:**

The optimization of endometrial receptivity (ER) through individualized therapies has been shown to enhance the likelihood of successful gestation. However, current practice lacks comprehensive methods for evaluating the ER of patients with recurrent pregnancy loss (RPL). Radiomics, an emerging AI-based technique that enables the extraction of mineable information from medical images, holds potential to offer a more objective and quantitative approach to ER assessment. This innovative tool may facilitate a deeper understanding of the endometrial environment and enable clinicians to optimize ER evaluation in RPL patients.

**Objective:**

This study aimed to identify ultrasound radiomics features associated with ER, with the purpose of predicting successful ongoing pregnancies in RPL patients, and to assess the predictive accuracy of these features against regular ER parameters.

**Methods:**

This retrospective, controlled study involved 262 patients with unexplained RPL and 273 controls with a history of uncomplicated full-term pregnancies. Radiomics features were extracted from ultrasound endometrial segmentation images to derive a radiomics score (rad-score) for each participant. Associations between rad-scores, baseline clinical variables, and sonographic data were evaluated using univariate and multivariate logistic regression analyses to identify potential indicators of RPL. Receiver operating characteristic (ROC) curve analysis was performed to evaluate the predictive accuracy of the rad-score and other identified indicators in discriminating RPL cases. Furthermore, the relationships between age and these identified indicators were assessed *via* Pearson correlation analysis.

**Results:**

From the 1312 extracted radiomics features, five non-zero coefficient radiomics signatures were identified as significantly associated with RPL, forming the basis of the rad-score. Following multivariate logistic regression analysis, age, spiral artery pulsatility index (SA-PI), vascularisation index (VI), and rad-score emerged as independent correlates of RPL (all P<0.05). ROC curve analyses revealed the superior discriminative capability of the rad-score (AUC=0.882) over age (AUC=0.778), SA-PI (AUC=0.771), and VI (AUC=0.595). There were notable correlations between age and rad-score (r=0.275), VI (r=-0.224), and SA-PI (r=0.211), indicating age-related variations in RPL predictors.

**Conclusion:**

This study revealed a significant association between unexplained RPL and elevated endometrial rad-scores during the WOI. Furthermore, it demonstrated the potential of rad-scores as a promising predictive tool for successful ongoing pregnancies in RPL patients.

## Introduction

Recurrent pregnancy loss (RPL) is a common complication, affecting approximately 3% of couples trying to conceive (1, 2). It is one of the most frustrating and difficult areas in reproductive medicine (3). Documented causes of this multifactorial disorder include chromosomal errors, immunological factors, anatomical defects, autoimmune disorders and endometrial dysfunction (4). However, despite recent advances, more than 50% of RPL women remain unidentified with current diagnostic modalities (5), which is called unexplained RPL (6).

Emerging evidence indicates that a dysregulated endometrial environment is associated with significant reproductive issues, notably RPL (7, 8). Implantation outside the period of endometrial receptivity (ER) called the window of implantation (WOI) has been reported to be closely related to early miscarriage (9, 10). Suboptimal ER is recognized to cause implantation failure, whereas it has been observed that RPL females may exhibit ‘superfertility’ - a phenomenon where an absence of selective mechanisms leads to the acceptance of abnormal embryos, thereby resulting in defective blastocyst implantation and seemingly normal pregnancies (11, 12).

Since ER can be improved with individualized therapies (13), assessing whether RPL women are at optimal ER through a deeper understanding of the endometrial environment may help in seeking the ideal balance of successful implantation and gestation. However, despite some progress, prevalent clinical practice predominantly relies on methodologies that lack precision in evaluating ER comprehensively for RPL patients. The existing studies have been primarily centered on endometrial parameters that are significant for predicting assisted reproductive outcomes, such as ultrasound-measured endometrial morphological attributes like thickness, volume, and pattern, as well as Doppler blood flow (14, 15). Yet, the reliability of these parameters as predictors for RPL is still under comprehensive analysis (16). Invasive procedures like hysteroscopy, despite offering detailed examination, are less suitable for repeated measurements (17). Advanced molecular tests, such as the endometrial receptivity array, show promise but still require extensive validation (18). Thus, the development of more comprehensive, non-invasive, and objective methodologies for ER assessment remains a critical focus in improving diagnostic precision and prognostic accuracy in RPL management.

More recently, radiomics is emerging as a cutting-edge method that uncovers hidden patterns and extracts a large amount of mineable information from medical images through the utilization of AI-based algorithms (19, 20). It holds significant potential for the prediction of live birth or ongoing pregnancy in unexplained RPL couples by providing a more objective and quantitative approach to ER assessment (21). Several investigations have looked into its potential in the endometrium, with the primary aim of diagnosing endometrial cancer (22–24). Despite this, its application in the field of reproductive medicine is limited.

The primary objective of this study was to investigate the ultrasound radiomics features that could differentiate ER in cases with RPL and healthy individuals in order to predict the probability of ongoing pregnancy in RPL patients. The secondary objective was to assess the predictive power of these key radiomics signatures in comparison to regular ER parameters, which were frequently used to judge subsequent live birth rates.

## Material and methods

### Patients

Between January 2019 and January 2022, a retrospective, controlled study was conducted at the First People’s Hospital of Wenling, enrolling a total of 600 female participants aged between 20 and 40 years. They included 300 patients with unexplained RPL and 300 women who had undergone ovulation tracking and achieved an uncomplicated full-term pregnancy without previous pregnancy loss. We defined unexplained RPL as ≥ 2 pregnancy losses before the 24th week of gestation (25), by exclusion of autoimmune, anatomic, genetic, endocrine, infectious factors, and male factors after a comprehensive investigation at their first visit.

The inclusion criteria for both groups were: 1) regular menstrual cycles with a duration between 27 to 35 days, 2) normal day-3 hormone profile, including follicle-stimulating hormone (FSH), luteinizing hormone (LH), and estradiol (E_2_), 3) normal ovarian and uterine ultrasonography, with no visible cysts, fibroids, polyps, or other noticeable structural abnormalities, 4) no use of hormonal contraceptives within the previous 3 months, 5) absence of prior gynecologic surgery, with the exception of procedures such as curettage, diagnostic laparoscopy, and hysteroscopy. In both groups, women were excluded if they had a history of smoking or heavy drinking, systemic diseases that might affect hemodynamic indexes, had taken steroid hormones, antibiotics, or medications such as aspirin or vitamin E that could potentially affect pregnancy

**Figure 1:**
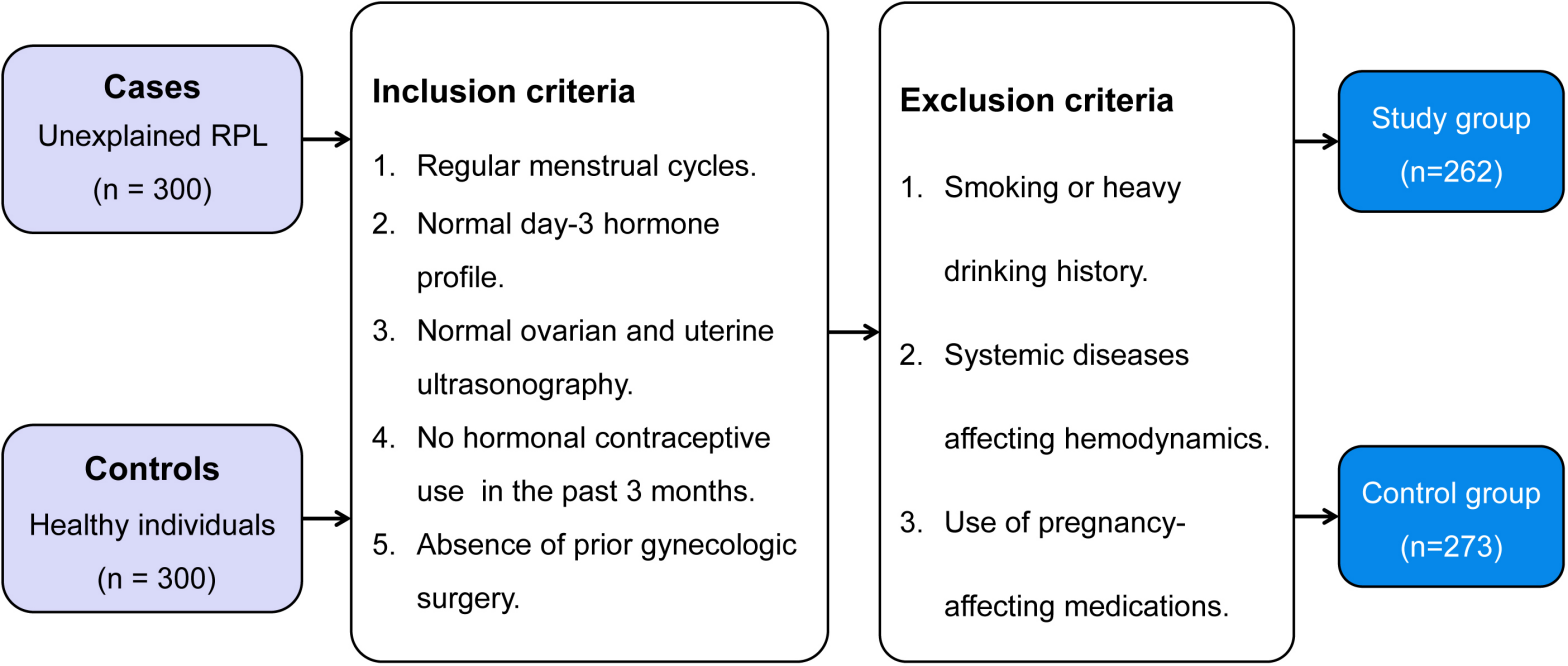
Selection process of the case-control study for patients with unexplained recurrent pregnancy loss and healthy individuals: a flowchart illustrating inclusion and exclusion criteria, leading to the composition of the study and control groups. RPL, recurrent pregnancy loss.

Ultimately, 262 patients with unexplained RPL were included and classified into the study group. Meanwhile, 283 healthy individuals made up the control group. **Figure 1** illustrates the detailed flowchart of the inclusion and exclusion process for the study participants. The present study was designed and performed with adherence to the Declaration of Helsinki and received approval from the Institutional Review Board of the First People’s Hospital of Wenling (KY-2022-1019-01). All women provided their informed consent, and to protect privacy, all collected data underwent anonymization prior to analysis.

### Data collection

Data were obtained during their initial consultation, which comprised of the following information: age, body mass index (BMI), number of prior miscarriages, day-3 hormone profile (FSH, LH, and E_2_) during the natural menstrual cycle, and antimüllerian hormone (AMH) rate.

### Transvaginal ultrasound

Transvaginal ultrasound scanning was implemented to all women during the WOI, 7 - 9 days post-ovulation with a standardized protocol. Ovulation was monitored using transvaginal ultrasound from the commencement of the menstrual cycle until confirmed. All imaging was performed by two experienced sonographers employing a Voluson™ E10 ultrasound system (GE, Boston, MA, USA) equipped with a RIC-9-D transvaginal volumetric probe and virtual organ computer-aided analysis (VOCAL) software.

Endometrial thickness (EMT) was determined in the longitudinal uterine plane, 2 cm from the uterine cavity base. A perpendicular line to the midline of the endometrium was drawn at the maximal distance between the anterior and posterior uterine myometrium interfaces. Three consecutive measurements were averaged for accuracy. Blood flow dynamics in the uterine arteries (UA) and spiral arteries (SA) were assessed non-invasively utilizing two-dimensional (2D) Doppler ultrasonography. This involved the calculation of the average values for the pulsatility index (PI) and resistance index (RI) from both the left and right UA and SA.

Three-dimensional (3D) mode was used to measure the endometrial volume, vascularization index (VI), flow index (FI), and vascularization flow index (VFI). We adjusted the volume of the sampling frame to encompass the full endometrium, using a volume angle of 120 degrees. VOCAL software was used to manually trace the endometrial outline in each section of a 30-degree aspect angle. After the outlines were completed, the system automatically calculated the volumetric results. The indices of vascularity were semiquantified within the defined area using the ‘histogram’ facility. Specifically, VI represented the ratio of power Doppler information, FI indicated the intensity of the power Doppler signal, and VFI integrated both aspects (26).

### Endometrial segmentation and radiomics feature extraction

Offline ultrasound images displaying the entire endometrium in a longitudinal section of the uterus were prepared for radiomics analyses. Utilizing 3D Slicer software (v5.0.2), the region of interest (ROI) containing the endometrium was manually traced by two trained sonographers who were blinded to the study. The endometrial segmentation images were then subjected to radiomics feature extraction. We leveraged PyRadiomics toolkit (v3.0.1) to extract a diverse range of features, encompassing six unique image types, seven feature classes, and wavelet transform.

### Feature selection and rad-score development

We utilized Z-score normalization to standardize all extracted features, thereby enabling compatibility with subsequent statistical analysis. The reliability of these features was evaluated by means of inter-operator reproducibility, which was utilized to determine the extent of agreement between different operators involved in the feature extraction process. Features demonstrating insufficient repeatability, as indicated by an interclass correlation coefficient (ICC) below 0.8, were excluded from further investigation. A Student’s t-test was utilized to compare each feature between unexplained RPL women and controls. To identify the features that are associated with unexplained RPL, a least absolute shrinkage and selection operator (LASSO) logistic regression was performed, with 10-fold cross validation to select the features related to unexplained RPL with nonzero coefficients from those having false discovery rate (FDR)-adjusted P values< 0.05 in the t-test.

**Figure 2:**
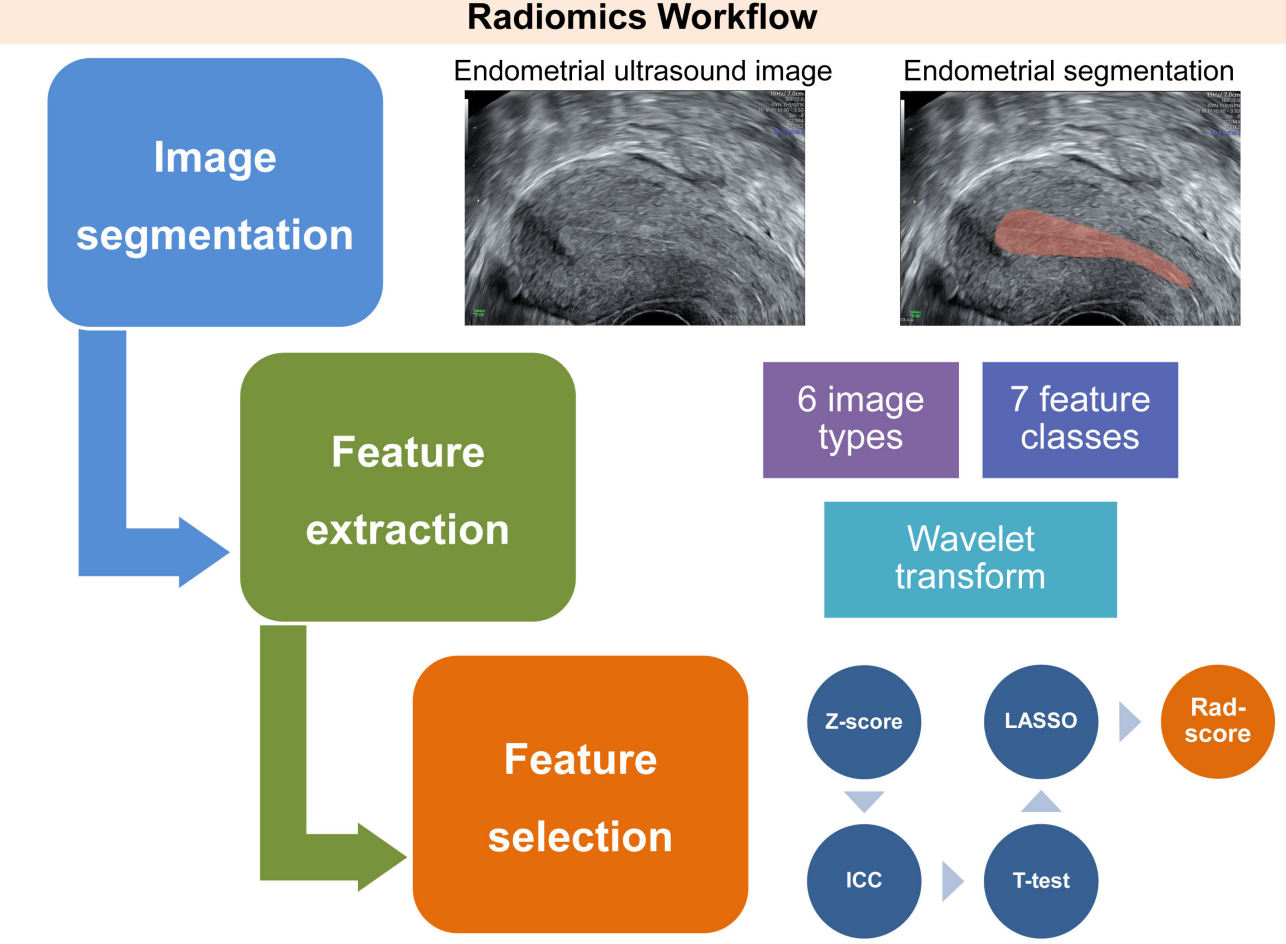
Workflow of three essential phases involved in the conception of an ultrasound-derived radiomics score, intended for the assessment of endometrial receptivity status. This process involves delineating a region of interest that encompasses the endometrium through a longitudinal section of the uterus. Subsequently, radiomics features are automatically extracted from six image types, seven feature classes, and wavelet transform, utilizing Pyradiomics, an open-source Python package. After normalization, the key radiomics features associated with unexplained recurrent pregnancy loss are identified by a three-step feature selection procedure. This procedure includes the interclass correlation coefficient test, Student’s t-test, and least absolute shrinkage and selection operator logistic regression. Finally, these features are integrated into the radiomics score utilizing linear regression. ICC, interclass correlation coefficient; LASSO, least absolute shrinkage and selection operator; Rad-score, radiomics score.

The computation of the radiomics score (rad-score) for each patient was achieved *via* a linear combination of the selected features, whereby the LASSO algorithm was utilized to assign weights. The workflow for conducting ultrasound endometrial radiomics analysis is presented in **Figure 2**, outlining the sequence of procedures employed in the analysis.

### Statistical analysis

The sample size was estimated using Power Analysis and Sample Size (PASS, v15.0). A review about RPL revealed that the incidence of inappropriate ER in RPL women was about 40% (P1 = 40%), whereas that in normal pregnant women was about 20% (P2 = 20%) (12). After setting the alpha level at 0.05 and the power at 0.9, through utilization of the PASS software, it has been determined that a minimum of 226 participants is necessary to achieve statistical significance. Accounting for a potential attrition rate of 10%, the study required a minimum of 251 participants in each group to achieve the necessary power for analysis.

To compare the differences in the data obtained between the study and control groups, statistical analyses were performed on both continuous and categorical variables. Normal distribution of the continuous variables was determined using the Shapiro-Wilk test, after which either an independent-sample t-test or a Mann-Whitney U test was applied. For the categorical variables, chi-square tests or Fisher’s exact tests were utilized, with the latter being applied when the expected count in any cell was less than five. Those variables that showed statistically significant differences between the groups were further analyzed using multivariate logistic regression analysis to ascertain the odds ratios (ORs) and corresponding 95% confidence intervals (CIs) for each independent correlate of unexplained RPL. Furthermore, receiver operating characteristic (ROC) curves were plotted and the area under the curve (AUC) was used for comparisons in different predictors through Delong’s test. A Pearson correlation analysis was performed to explore the potential relationship between the identified ER indices and the age of patients. Data processing and analysis were performed using IBM SPSS Statistics (v 22.0, SPSS Inc.), MedCalc (v 19.2.1), R package (v 4.2.1), and Python (v 3.7.1). For all conducted statistical tests, we regarded a p-value< 0.05 as the threshold for defining statistical significance.

## Results

### Radiomics analyses

In the present study, we extracted and normalized the radiomics features from the endometrial ultrasound image of each participant and investigate their potential association with RPL. A total of 1312 radiomics features were extracted, and 83.9% (1102 features) were chosen for further investigation since their intra-observer ICC values were greater than 0.8. Student’s t-tests were performed on these features, and 69 with FDR-adjusted *P* values< 0.05 were initially screened for further analysis.

**Figure 3:**
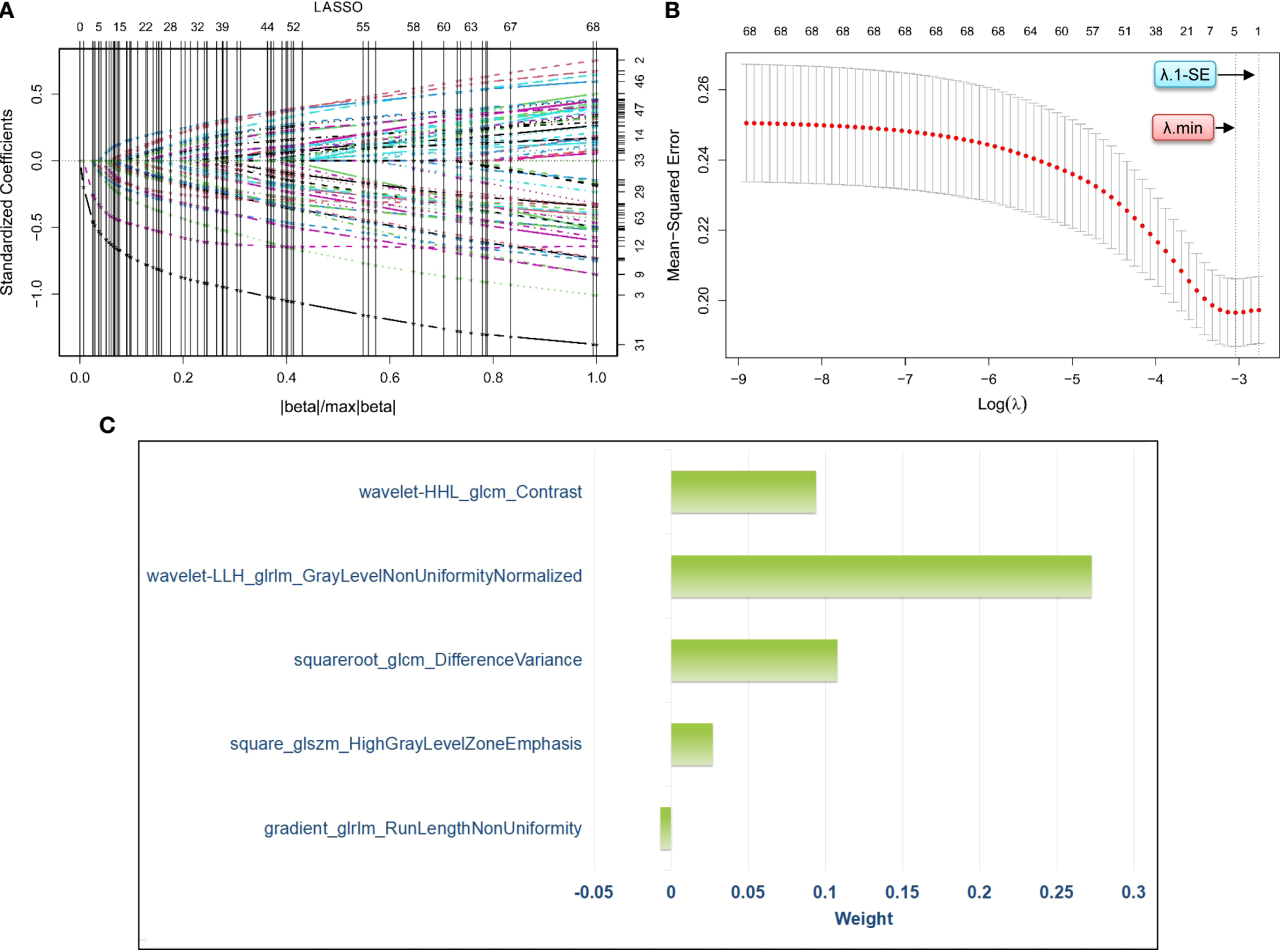
Process of radiomics feature selection *via* least absolute shrinkage and selection operator logistic regression for the development of a radiomics score. Specifically, **(A)** visualizes the distribution of coefficients from the least absolute shrinkage and selection operator for the 69 radiomics features pre-screened by the Student’s t-test. **(B)** shows the optimal selection process of the penalty regularization parameter, which involved 10-fold cross-validation using both the minimum criteria and the 1-standard error of the minimum criteria. **(C)** presents the weights assigned to the five selected radiomics signatures with nonzero coefficients, which are determined based on the optimal penalty regularization parameter identified using the minimum criteria in the method of least absolute shrinkage and selection operator. LASSO, least absolute shrinkage and selection operator; λ, penalty regularization parameter; λ.min, minimum criteria, λ;1-SE, 1-standard error of the minimum criteria.

We developed a penalty function and chose the optimal penalty regularization parameter (λ) with the minimum criteria in the LASSO model to identify the radiomics signatures associated with RPL (**Figures 3A, B**). Using this method, we were able to choose five radiomics signatures with nonzero coefficients that were found to be associated with RPL (**Figure 3C**). These signatures and their coefficients served as the foundation for the rad-score. The detailed calculation of the rad-score is presented in **Supplemental Appendix 1**.

### Clinical and ultrasound data of study women

**Table 1** presents the rad-score, as well as baseline clinical and ultrasound data for both groups. Upon their first visit, patients in the study group experienced 2, 3, and 4 or more spontaneous miscarriages, accounting for 52.3%, 34.0%, and 13.7%, respectively. The distribution of gestational age at miscarriage revealed that 346 cases (50.1%) occurred before the 6th week, 251 cases (36.4%) between the 6th and 12th weeks, and 93 cases (13.5%) after the 12th week. No differences were found between the groups with respect to BMI, day-3 hormone profile, AMH rate, and ultrasound parameters except SA-PI, SA-RI, and VI (all *P* values > 0.05). Patients with unexplained RPL demonstrated a significant increase in age, SA-PI, SA-RI, and rad-score compared to the controls (all *P* values< 0.05). Furthermore, women with unexplained RPL had a lower VI in comparison to the control group (P = 0.023).

**Table 1:** Comparison of the rad-score and multiple clinical characteristics among women in the study and control groups.

Variable		Study group (n=262)	Control group (n=273)	*P* value
Age, year		35 (33–37)	32 (29–34)	<0.001^*^
BMI, kg/m^2^		21.42 ± 3.14	21.72 ± 3.66	0.321^#^
Number of miscarriages, n (%)	2	137 (52.3%)	–	–
	3	89 (34.0%)	–	
	≥4	36 (13.7%)	–	
Day-3 hormone profile	FSH (IU/L)	7.32 ± 1.47	7.45 ± 1.38	0.381^#^
	LH (IU/L)	6.77 ± 0.96	6.52 ± 0.87	0.528^#^
	E_2_ (pg/mL)	34.8 ± 5.9	33.5 ± 5.7	0.633^#^
AMH, ng/ml		1.45 ± 0.40	1.50 ± 0.42	0.161
Ultrasound parameters	EMT, mm	8.80 ± 1.78	9.02 ± 1.81	0.150^#^
	SA-PI	1.12 (0.95-1.26)	0.92 (0.83-1.01)	<0.001^*^
	SA-RI	0.55 (0.52-0.60)	0.53 (0.50-0.56)	0.018^*^
	UA-PI	2.12 (2.01-2.26)	2.10 (1.98-2.23)	0.151^*^
	UA-RI	0.83 (0.80-0.85)	0.82 (0.80-0.84)	0.132^*^
	Endometrial volume, ml	4.87 ± 0.82	5.01 ± 1.06	0.101^#^
	VI, %	2.29 (1.92-2.69)	2.41 (2.11-2.83)	0.023^*^
	FI	25.9 (21.2-32.0)	26.5 (23.4-31.0)	0.247^*^
	VFI	0.557 (0.427-0.739)	0.645 (0.503-0.813)	0.164^*^
Rad-score		0.483 (0.402-0.565)	0.282 (0.219-0.370)	<0.001^*^

*for Mann-Whitney U test and ^#^for independent sample t-test. BMI, body mass index; FSH, follicle-stimulating hormone; LH, luteinizing hormone; E_2_, estradiol; AMH, antimüllerian hormone; EMT, endometrial thickness; SA, spiral artery; UA, uterine artery; PI, pulsatility index; RI, resistance index; VI, vascularisation index; FI, flow index; VFI, vascularisation flow index; Rad-score, radiomics score.

### Independent correlates of unexplained RPL

Univariate and multivariate logistic regression analyses were conducted to determine the independent correlates of unexplained RPL, as indicated in **Table 2**. The variables considered in the analysis were derived from indicators with statistically significant differences between the two groups. The multivariate analysis revealed that age, SA-PI, VI, and rad-score were identified as independent determinants of unexplained RPL. Conversely, SA-RI did not exhibit a significant association with unexplained RPL. These findings suggested that rad-score, along with age and SA blood flow indicators, could serve as predictors of RPL.

**Table 2:** Univariate and multivariate logistic regression analyses for the independent correlates of unexplained RPL.

Variable	Univariate regression		Multivariate regression	
	OR (95% CI)	*P*	OR (95% CI)	*P*
Age	1.428 (1.332-1.531)	<0.001	1.533 (1.369-1.718)	<0.001
VI	0.552 (0.409-0.744)	<0.001	0.405 (0.237-0.692)	0.001
SA-PI	1.827^*^ (1.620-2.062)	<0.001	1.869^*^ (1.563-2.236)	<0.001
SA-RI	2.118^*^ (1.490-3.008)	<0.001	1.713^*^ (0.952-3.080)	0.072
Rad-score	1.157^**^ (1.129-1.186)	<0.001	1.161^**^ (1.125-1.197)	<0.001

The OR values represented by * is the elevated risk per 0.1-unit increment, and the ** represents the elevated risk per 0.01-unit increment. OR, odds ratio; CI, confidence interval; SA, spiral artery; PI, pulsatility index; RI, resistance index; VI, vascularisation index; Rad-score, radiomics score.

### Comparison of different predictors of RPL

**Figure 4:**
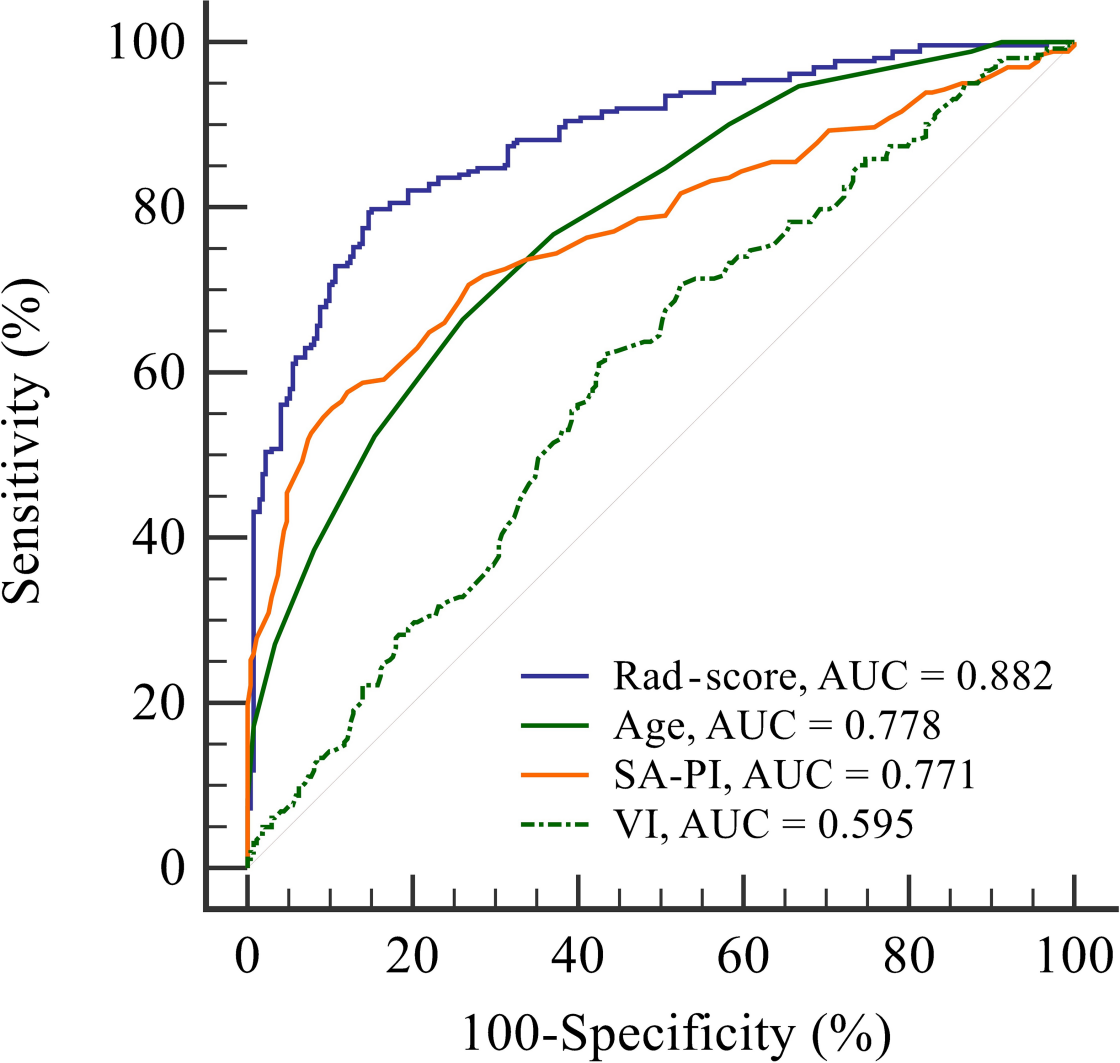
Receiver operating characteristic curve analysis to evaluate the discriminatory power of four indicators: radiomics score, age, spiral artery pulsatility index, and vascularisation index for predicting recurrent pregnancy loss. The radiomics score shows the highest area under the curve value of 0.882, indicating its superior performance in identifying individuals with recurrent pregnancy loss compared to the other indicators. AUC, area under the curve; Rad-score, radiomics score; SA-PI, spiral artery pulsatility index; VI, vascularisation index.

ROC curve analyses were conducted to assess the predictive capabilities of the four indicators (rad-score, age, SA-PI, and VI) for RPL (**Figure 4**). Their AUC values were determined to be 0.882, 0.778, 0.771, and 0.595, respectively. It indicated that rad-score exhibited the strongest discriminative power among the indicators in identifying individuals with RPL and showed promising potential for RPL prediction.

### Correlation analysis with age

**Figure 5:**
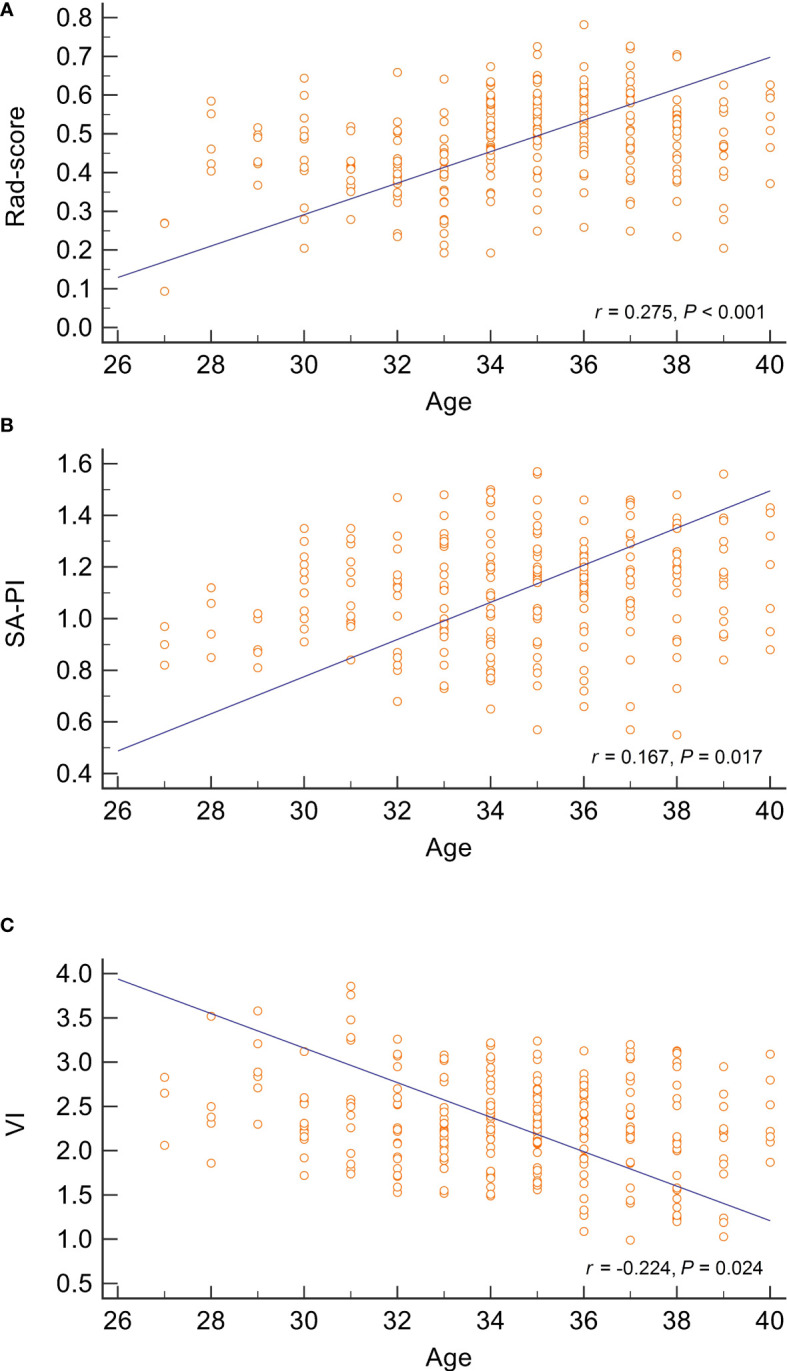
Correlation analysis between age and three predictors of recurrent pregnancy loss. The scatter plots represent the relationship between age and radiomics score, spiral artery pulsatility index, and vascularisation index, as displayed in **(A–C)**, respectively. The analysis suggests a positive correlation between age and both radiomics score and spiral artery pulsatility index, while age is inversely correlated with the vascularisation index. SA-PI, spiral artery pulsatility index,;VI, vascularisation index; Rad-score, radiomics score.

To investigate the potential associations between age and these RPL predictors, correlation analyses were performed for patients in the study group. The correlation coefficients for rad-score, SA-PI, and VI were 0.275, 0.211, and -0.224, respectively (all P values< 0.05). The scatterplot in **Figure 5** revealed a negative correlation between endometrial blood perfusion and age, as well as a positive correlation between rad-score and age. It indicated that the endometrial radiomics signatures in RPL patients, which are not visually detectable, were also age-related.

## Discussion

This study investigated the potential of ultrasound radiomics signatures to distinguish unexplained RPL patients from healthy individuals. Rad-score, a parameter calculated from radiomics of endometrial ultrasound images, was similar to traditional ER parameters, showing age-related variation. The ROC curves revealed the superior discriminatory ability of the rad-score, compared to other indicators (age, SA-PI, and VI), in identifying RPL patients. With further validation, radiomics analysis may contribute to a more precise assessment of ER in RPL patients. This could subsequently facilitate the implementation of targeted interventions to improve the prognostic outcomes associated with pregnancy in these patients

The successful initiation of pregnancy depends on the effective implantation of an embryo within a receptive endometrium. RPL remains a multifaceted condition, with nearly half of all cases classified as unexplained, often attributed to non-chromosomal uterine factors (27–29). Current research indicates a potential association between RPL and a deficient differentiation process in endometrial stromal cells, leading to a shortage of specialized decidual cells. This functional deficit in decidualization increases the risk of delayed implantation, suboptimal embryo quality control, and early placental failure, regardless of the embryo’s karyotype (30). Notably, a phenomenon known as “superfertility” has been identified, where patients who exhibit exceptional fertility often conceive within one or two menstrual cycles, but subsequently experience miscarriage due to the endometrium’s inability to recognize, respond to, and eliminate impaired embryos (12).

It has been observed that women with superfertility manifest a prolonged WOI, resulting in reduced endometrial perfusion within 7-9 days after ovulation. This deficiency, characterized by increased vascular resistance and impaired blood flow distribution (31, 32), is reflected in our findings *via* increased SA-PI and reduced VI among RPL patients versus the control group. In our study, we observed a similarity in endometrial morphology - particularly with respect to thickness and volume - between the study and control groups. This observation might seem counterintuitive given the role of the endometrium in pregnancy. However, it aligns with previous literature indicating an inconsistent correlation between endometrial thickness, hormone levels, and pregnancy outcomes (33, 34). Certain studies have suggested that endometrial morphology captured by 2D ultrasound may not serve as a definitive predictor of early spontaneous miscarriage or pregnancy rates, indicating that more complex mechanisms may be involved (30, 32, 35).

Despite this, our study revealed that there were significant differences in radiomics features extracted from endometrial ultrasound images between the two groups. Radiomics features are a set of mathematically-derived parameters that are capable of indicating intra-region heterogeneity, aiding in clinical diagnosis and prognostic assessment (36). These features have gained significant attention in recent years due to their ability to provide a non-invasive approach for capturing microcosmic details that may be challenging to assess through visual interpretation (37). In this study, for the first time, we have utilized these radiomics features to analyze the ultrasound signatures of patients with unexplained RPL. The rad-score, derived from these features, demonstrated superior performance in identifying unexplained RPL patients when compared to conventional ER indicators. This finding suggests that radiomics, with its comprehensive view of the endometrium, offers valuable insights into its intricate and multifaceted characteristics that extend beyond those captured by single parameters. In particular, the radiomics feature “wavelet-LLH_glrlm_GrayLevelNonUniformityNormalized” displayed the strongest association with RPL, indicating the significance of these novel parameters in deepening our understanding of ER. Our findings suggest that radiomics features might enable more precise and comprehensive assessments of ER. This novel method could potentially bridge existing knowledge gaps in the etiology of unexplained RPL. By extracting extensive information regarding the endometrium, radiomics may enhance our understanding of ER, thereby potentially contributing to the optimization of diagnostic and therapeutic approaches for RPL.

The identification and validation of radiomics features predictive of RPL marks a significant step towards potential therapeutic interventions and preventive measures. Utilizing the unique strengths of radiomics, such as scalability and rapid validation, these approaches could be efficiently implemented and broadly applied (38). Specifically, radiomics can provide a comprehensive assessment of ER, enabling tailored interventions to optimize the endometrial environment for pregnancy. Clinicians could then monitor these radiomics scores, adjusting therapeutic strategies in real time to enhance ER and subsequently increase the likelihood of successful pregnancies. Moreover, the wide-ranging applicability of radiomics could encourage widespread, multicenter studies, aimed at further refining and validating treatment strategies based on radiomics features. This strategy, in line with the principles of precision medicine, holds promise for enhancing prognostic outcomes for RPL patients and deepening our understanding of the complex nature of RPL.

To ensure a comprehensive evaluation of the study, it is necessary to address several limitations. Firstly, due to the retrospective nature, only the number of previous miscarriages at the initial visit was recorded, and an age-matched case-control study was not implemented. This not only prevented an evaluation of the correlation between the number of miscarriages and ER data, but also left unexplored the potential impact of age differences, which might influence other age-related indicators, on the reproductive outcome in RPL group. Secondly, the case-control study design restricted the analysis to the causes of RPL and did not allow for the prediction of the subsequent pregnancy success rate of RPL patients. Thirdly, while interobserver reproducibility was considered, the study was conducted at a single medical center using a specific ultrasound scanner. This limitation might potentially lead to inter-observer variability due to differences in ultrasound machines or operators, which may impact the performance of radiomics analyses. Finally, the clinical application of radiomics is still in its early stages, and caution is required when interpreting radiomic features (39). Furthermore, the reproducibility and validation of various radiomics techniques have yet to be standardized, and alterations at any stage may impact the features and ultimate output. Future investigations should strive to overcome current limitations by conducting larger, age-matched, prospective cohort studies across multiple medical centers, while also considering the implementation of an inter-rater reliability test to assess consistency among multiple ultrasound readings. These endeavors will be critical in confirming the role of rad-scores as reliable ER markers in unexplained RPL patients.

## Conclusion

The present study revealed a significant association between unexplained RPL and increased endometrial rad-scores during the WOI, highlighting the potential of suboptimal ER as a significant factor in these cases. Our findings indicate the promise of rad-scores as a tool for predicting the likelihood of ongoing pregnancy in RPL patients, potentially enhancing the array of currently employed ER parameters.

## Data availability statement

The original contributions presented in the study are included in the article/**Supplementary Material**. Further inquiries can be directed to the corresponding author.

## Ethics statement

The studies involving human participants were reviewed and approved by Institutional Review Boards of the First People’s Hospital of Wenling (KY-2022-1019-01). The patients/participants provided their written informed consent to participate in this study.

## Author contributions

Study design: WH and ML. Data collection and analysis: WH, YJ, LJ, and ML. Supervision: WH and ML. Statistics: WH, YJ, and LJ. Manuscript writing: WH, YJ, LJ, and ML. Manuscript revision: WH and ML. All authors contributed to the article and approved the submitted version.

## Supplementary material

Supplementary Appendix 1 Rad-score = 0.5724616-0.006894×gradient_glrlm_RunLengthNonUniformity

## References

[1] Garrido-Gimenez C Alijotas-Reig J. Recurrent miscarriage: causes, evaluation and management. Postgrad Med J (2015) 91(1073):151–62. doi: 10.1136/postgradmedj-2014-132672 25681385

[2] Larsen EC Christiansen OB Kolte AM Macklon N. New insights into mechanisms behind miscarriage. BMC Med (2013) 11:154. doi: 10.1186/1741-7015-11-154 23803387 PMC3699442

[3] Lejeune V. Recurrent spontaneous miscarriage: etiology, and management of subsequent pregnancies. J Gynecol Obstet Biol Reprod (Paris) (2010) 39(3 Suppl):F11–6. doi: 10.1016/j.jgyn.2010.02.012 20356688

[4] Dimitriadis E Menkhorst E Saito S Kutteh WH Brosens JJ. Recurrent pregnancy loss. Nat Rev Dis Primers (2020) 6(1):98. doi: 10.1038/s41572-020-00228-z 33303732

[5] Ticconi C Pietropolli A Di Simone N Piccione E Fazleabas A. Endometrial immune dysfunction in recurrent pregnancy loss. Int J Mol Sci (2019) 20(21):5332. doi: 10.3390/ijms20215332 31717776 PMC6862690

[6] Rai R Regan L. Recurrent miscarriage. Lancet (2006) 368(9535):601–11. doi: 10.1016/s0140-6736(06)69204-0 16905025

[7] Russell P Anderson L Lieberman D Tremellen K Yilmaz H Cheerala B. The distribution of immune cells and macrophages in the endometrium of women with recurrent reproductive failure I: Techniques. J Reprod Immunol (2011) 91(1-2):90–102. doi: 10.1016/j.jri.2011.03.013 21783262

[8] Kuon R-J Weber M Heger J Santillán I Vomstein K Bär C. Uterine natural killer cells in patients with idiopathic recurrent miscarriage. Am J Reprod Immunol (2017) 78(4):e12721. doi: 10.1111/aji.12721 28639334

[9] Banerjee P Ghosh S Dutta M Subramani E Khalpada J RoyChoudhury S. Identification of key contributory factors responsible for vascular dysfunction in idiopathic recurrent spontaneous miscarriage. PloS One (2013) 8(11):e80940. doi: 10.1371/journal.pone.0080940 24260517 PMC3829935

[10] Lessey BA Young SL. What exactly is endometrial receptivity? Fertil Steril (2019) 111(4):611–7. doi: 10.1016/j.fertnstert.2019.02.009 30929718

[11] Salker M Teklenburg G Molokhia M Lavery S Trew G Aojanepong T. Natural selection of human embryos: impaired decidualization of endometrium disables embryo-maternal interactions and causes recurrent pregnancy loss. PloS One (2010) 5(4):e10287. doi: 10.1371/journal.pone.0010287 20422017 PMC2858209

[12] Teklenburg G Salker M Heijnen C Macklon NS Brosens JJ. The molecular basis of recurrent pregnancy loss: impaired natural embryo selection. Mol Hum Reprod (2010) 16(12):886–95. doi: 10.1093/molehr/gaq079 20847090

[13] Makrigiannakis A Makrygiannakis F Vrekoussis T. Approaches to improve endometrial receptivity in case of repeated implantation failures. Front Cell Dev Biol (2021) 9:613277. doi: 10.3389/fcell.2021.613277 33796523 PMC8007915

[14] Casper RF Yanushpolsky EH. Optimal endometrial preparation for frozen embryo transfer cycles: window of implantation and progesterone support. Fertil Steril (2016) 105(4):867–72. doi: 10.1016/j.fertnstert.2016.01.006 26820769

[15] Paulson RJ. Introduction: Endometrial receptivity: evaluation, induction and inhibition. Fertil Steril (2019) 111(4):609–10. doi: 10.1016/j.fertnstert.2019.02.029 30929717

[16] Craciunas L Gallos I Chu J Bourne T Quenby S Brosens JJ. Conventional and modern markers of endometrial receptivity: a systematic review and meta-analysis. Hum Reprod Update (2019) 25(2):202–23. doi: 10.1093/humupd/dmy044 30624659

[17] Bailey AP Jaslow CR Kutteh WH. Minimally invasive surgical options for congenital and acquired uterine factors associated with recurrent pregnancy loss. Womens Health (Lond) (2015) 11(2):161–7. doi: 10.2217/whe.14.81 25776290

[18] Patel BG Lessey BA. Clinical assessment and management of the endometrium in recurrent early pregnancy loss. Semin Reprod Med (2011) 29(6):491–506. doi: 10.1055/s-0031-1293203 22161462

[19] Gillies RJ Kinahan PE Hricak H. Radiomics: images are more than pictures, they are data. Radiology (2016) 278(2):563–77. doi: 10.1148/radiol.2015151169 PMC4734157 26579733

[20] Rizzo S Botta F Raimondi S Origgi D Fanciullo C Morganti AG. Radiomics: the facts and the challenges of image analysis. Eur Radiol Exp (2018) 2(1):36. doi: 10.1186/s41747-018-0068-z 30426318 PMC6234198

[21] Khamisy-Farah R Furstenau LB Kong JD Wu J Bragazzi NL. Gynecology meets big data in the disruptive innovation medical era: state-of-art and future prospects. Int J Environ Res Public Health (2021) 18(10):5058. doi: 10.3390/ijerph18105058 34064710 PMC8151939

[22] Bogani G Chiappa V Lopez S Salvatore C Interlenghi M D’Oria O. Radiomics and molecular classification in endometrial cancer (The ROME study): A step forward to a simplified precision medicine. Healthcare (2022) 10(12):2464. doi: 10.3390/healthcare10122464 36553988 PMC9778151

[23] Jin X Ai Y Zhang J Zhu H Jin J Teng Y. Noninvasive prediction of lymph node status for patients with early-stage cervical cancer based on radiomics features from ultrasound images. Eur Radiol (2020) 30(7):4117–24. doi: 10.1007/s00330-020-06692-1 32078013

[24] Manganaro L Nicolino GM Dolciami M Martorana F Stathis A Colombo I. Radiomics in cervical and endometrial cancer. Br J Radiol (2021) 94(1125):20201314. doi: 10.1259/bjr.20201314 34233456 PMC9327743

[25] Group. EEPGD. Recurrent pregnancy loss: guideline of the European Society of Human Reproduction and Embryology . Available at: https://www.eshre.eu/Guidelines-and-Legal/Guidelines/Recurrent-pregnancy-loss.aspx.

[26] Raine-Fenning NJ Campbell BK Clewes JS Kendall NR Johnson IR. The reliability of virtual organ computer-aided analysis (VOCAL) for the semiquantification of ovarian, endometrial and subendometrial perfusion. Ultrasound Obstet Gynecol (2003) 22(6):633–9. doi: 10.1002/uog.923 14689538

[27] Alijotas-Reig J Garrido-Gimenez C. Current concepts and new trends in the diagnosis and management of recurrent miscarriage. Obstet Gynecol Surv (2013) 68(6):445–66. doi: 10.1097/OGX.0b013e31828aca19 23942472

[28] Practice Committee of the American Society for Reproductive Medicine. Evaluation and treatment of recurrent pregnancy loss: a committee opinion. Fertil Steril (2012) 98(5):1103–11. doi: 10.1016/j.fertnstert.2012.06.048 22835448

[29] van Dijk MM Kolte AM Limpens J Kirk E Quenby S van Wely M. Recurrent pregnancy loss: diagnostic workup after two or three pregnancy losses? A systematic review of the literature and meta-analysis. Hum Reprod Update (2020) 26(3):356–67. doi: 10.1093/humupd/dmz048 PMC7161667 32103270

[30] Pirtea P Cicinelli E De Nola R de Ziegler D Ayoubi JM. Endometrial causes of recurrent pregnancy losses: endometriosis, adeno myosis, and chronic endometritis. Fertil Steril (2021) 115(3):546–60. doi: 10.1016/j.fertnstert.2020.12.010 33581856

[31] Velauthar L Plana MN Kalidindi M Zamora J Thilaganathan B Illanes SE. First-trimester uterine artery Doppler and adverse pregnancy outcome: a meta-analysis involving 55,974 women. Ultrasound Obstet Gynecol (2014) 43(5):500–7. doi: 10.1002/uog.13275 24339044

[32] Tan SY Hang F Purvarshi G Li MQ Meng DH Huang LL. Decreased endometrial vascularity and receptivity in unexplained recurrent miscarriage patients during midluteal and early pregnancy phases. Taiwan J Obstet Gynecol (2015) 54(5):522–6. doi: 10.1016/j.tjog.2014.10.008 26522103

[33] Rabinowitz R Laufer N Lewin A Navot D Bar I Margalioth EJ. The value of ultrasonographic endometrial measurement in the prediction of pregnancy following in *vitro* fertilization. Fertil Steril (1986) 45(6):824–8. doi: 10.1016/s0015-0282(16)49400-8 3086131

[34] Sterzik K Grab D Schneider V Strehler EJ Gagsteiger F Rosenbusch BE. Lack of correlation between ultrasonography and histologic staging of the endometrium in *in vitro* fertilization (IVF) patients. Ultrasound Med Biol (1997) 23(2):165–70. doi: 10.1016/s0301-5629(96)00197-4 9140174

[35] Carbonnel M Pirtea P de Ziegler D Ayoubi JM. Uterine factors in recurrent pregnancy losses. Fertil Steril (2021) 115(3):538–45. doi: 10.1016/j.fertnstert.2020.12.003 33712099

[36] Abbasian Ardakani A Bureau NJ Ciaccio EJ Acharya UR. Interpretation of radiomics features-A pictorial review. Comput Methods Programs Biomed (2022) 215:106609. doi: 10.1016/j.cmpb.2021.106609 34990929

[37] Mayerhoefer ME Materka A Langs G Häggström I Szczypiński P Gibbs P. Introduction to radiomics. J Nucl Med (2020) 61(4):488–95. doi: 10.2967/jnumed.118.222893 PMC9374044 32060219

[38] Guiot J Vaidyanathan A Deprez L Zerka F Danthine D Frix A-N. A review in radiomics: Making personalized medicine a reality *via* rout ine imaging. Med Res Rev (2022) 42(1):426–40. doi: 10.1002/med.21846 34309893

[39] Rutman AM Kuo MD. Radiogenomics: creating a link between molecular diagnostics and diagnostic imaging. Eur J Radiol (2009) 70(2):232–41. doi: 10.1016/j.ejrad.2009.01.050 19303233

