# Supplementary material for: Radiomics optimizing the evaluation of endometrial receptivity for women with unexplained recurrent pregnancy loss
Source: Front Endocrinol (Lausanne). 2023 Aug 8;14:1181058. doi: 10.3389/fendo.2023.1181058 (PMC10545880; doi:10.3389/fendo.2023.1181058)
Supplement: Supplementary Appendix 1 — Rad-score = 0.5724616-0.006894×gradient_glrlm_RunLengthNonUniformity [file Table_1.docx]

**Supplementary**

**Appendix 1**

Rad-score = 0.5724616-0.006894×gradient_glrlm_RunLengthNonUniformity

+0.027095×square_glszm_HighGrayLevelZoneEmphasis

+0.107855×squareroot_glcm_DifferenceVariance

+0.272675×wavelet-LLH_glrlm_GrayLevelNonUniformityNormalized

+0.093734×wavelet-HHL_glcm_Contrast
